# Light Adjustable Lens (LAL) using Yamane intrascleral flanged fixation for traumatic aphakia: A case report

**DOI:** 10.1016/j.ajoc.2026.102663

**Published:** 2026-09-16

**Authors:** Korolos Sawires, Dana Toameh, Raj Maini, Sohel Somani

**Affiliations:** a Faculty of Medicine, Dalhousie University, Halifax, Nova Scotia, Canada; b Faculty of Medicine, McGill University, Montreal, Quebec, Canada; c U Vision Group, Brampton, Ontario, Canada; d North York General Hospital, North York, Ontario, Canada; e William Osler Health Systems, Brampton, Ontario, Canada; f Department of Ophthalmology & Vision Sciences, University of Toronto, Toronto, Ontario, Canada

**Keywords:** Light adjustable lens, Yamane technique, Scleral fixation, Intraocular lens, Aphakia, Case report

## Abstract

**Purpose:**

To describe the novel use of a Light Adjustable Lens (LAL) implanted using Yamane flanged intrascleral fixation for the management of post-traumatic aphakia without capsular support.

**Observations:**

A 60-year-old male presented with aphakia in the right eye following remote ocular trauma during adolescence. Preoperative corrected distance visual acuity was 20/70 with a hyperopic refraction of +9.00 + 3.50 × 87. Biometry revealed high corneal astigmatism (4.43 diopters) and an axial length of 23.52 mm. In the absence of capsular support, a +19.5 D LAL was implanted using the Yamane double-needle flanged intrascleral fixation technique. Postoperatively, the patient underwent four light-delivery treatments over 14 weeks. By week 14, corrected distance visual acuity improved to 20/40−2 with uncorrected distance visual acuity of 20/70−2. Final manifest refraction was 0.00 −2.50 × 180, achieving excellent binocular alignment with the fellow eye. The lens remained well centered throughout follow-up, with no intraoperative or postoperative complications such as cystoid macular edema or elevated intraocular pressure.

**Conclusions and importance:**

This case demonstrates the feasibility of combining Yamane flanged intrascleral fixation with the LAL in post-traumatic aphakia, providing both stable fixation and the unique advantage of postoperative refractive adjustment. In contrast to the refractive unpredictability typically associated with secondary intraocular lens implantation, the LAL enabled precise refinement of the final refractive outcome. These findings highlight the potential role of the LAL in expanding the refractive precision of secondary intraocular lens surgery, particularly in anatomically complex eyes.

## 1 Introduction

Management of aphakia in the absence of capsular support poses a significant surgical challenge, particularly following complicated cataract extraction, trauma, or late intraocular lens (IOL) dislocation. In these cases, options for visual rehabilitation include angle-supported anterior chamber IOLs (ASAC-IOLs), iris-fixated IOLs (IF-IOLs), and scleral-fixated IOLs (SF-IOL), each associated with distinct risk considerations.^1^^,^^2^ ASAC-IOLs are known to confer increased risks of corneal decompensation, uveitis, glaucoma, and chronic endothelial cell loss, especially in eyes with pre-existing corneal pathology or shallow anterior chambers.^1^^,^^2^ IF-IOLs are less suitable when the iris tissue is compromised, given the elevated risk of chronic inflammation, pigment dispersion, and secondary glaucoma.^2^^,^^3^ Although sutured SF-IOLs remain a standard option, long term risks such as suture degradation, late IOL dislocation, and suture-related complications limit their use, especially in younger or active patients.^1^^,^^2^

To address these limitations, sutureless flanged intrascleral fixation, exemplified by the Yamane technique, has emerged as a significant advancement. This technique eliminates the need for sutures by externalizing and flanging the haptics of a three-piece IOL within the sclera, resulting in stable and well-centered lens fixation with lower rates of late dislocation and fewer anterior segment complications.^4^, ^5^, ^6^ The method also preserves normal ocular anatomy and minimizes manipulation of already compromised tissues, making it especially advantageous in complex cases.

Despite these technical advances, predicting effective lens position and achieving desired refractive outcomes remain challenging in secondary IOL procedures. Without a capsular bag, lens centration is less predictable, leading to higher rates of postoperative refractive error, tilt, and decentration compared with primary in the bag implantation.^5^^,^^6^ Among available three-piece IOLs for flanged fixation, certain modern lens designs are noted for their favorable haptic properties and stability.^3^

The Light Adjustable Lens (LAL, RxSight, Aliso Viejo, CA) is a three-piece photoreactive silicone intraocular lens containing photosensitive macromers, allowing postoperative adjustment of lens power through targeted ultraviolet light treatment (LDD). Targeted LDD induces polymerization of macromers within the irradiated regions of the lens. This creates a concentration gradient that drives diffusion of unpolymerized macromers, resulting in redistribution of material and predictable changes in lens curvature and refractive power. By varying the spatial pattern and energy of ultraviolet exposure, both spherical and cylindrical adjustments can be achieved in a controlled, dose-dependent manner. Once the desired refractive outcome is obtained, a final LDD lock in treatment polymerizes the remaining macromers preventing further change.^7^ LAL is designed for implantation within the capsular bag and has demonstrated excellent refractive precision in standard cataract cases, with the majority of patients achieving their target refraction.^8^ While LAL is FDA approved for in-the-bag use, studies have suggested the LAL haptics possess sufficient tensile strength and durability for flanged fixation.^8^ Despite these findings, clinical experience with LAL implantation via Yamane fixation is extremely limited, with only two cases reported in the literature.^4^^,^^5^

We hypothesized that Yamane intrascleral fixation of the LAL would combine stable lens placement with the unique advantage of postoperative refractive adjustment. While LAL implantation with intrascleral fixation has been reported in ectopia lentis and Marfan syndrome, its use in post-traumatic aphakia has not been well characterized. Here, we present a case of post-traumatic aphakia with no existing capsular or zonular support, managed with LAL implantation via Yamane flanged intrascleral fixation, and summarize the rationale, surgical approach, and early postoperative outcomes.

## 2 Case report

A 60-year-old male patient presented to our institute and was referred for management of aphakia in the right eye (OD) following remote ocular trauma. As an early teen, he had sustained a blunt trauma that led to a ruptured lens capsule. The lens was removed at that time without primary IOL implantation.

Preoperative uncorrected distance visual acuity (UDVA) was counting fingers (CF) at 4 feet OD and 20/30– 2 in the left eye (OS). Autorefraction and keratometry revealed a steep corneal profile of 47.50 diopters (D) OD, a corneal astigmatism of +4.43 D, and a highly hyperopic aphakic refraction of +9.00 + 3.50 × 87. Autorefraction was plano −0.5 x 165 OS with a corrected distance visual acuity (CDVA) of 20/20. Manifest refraction OD showed +1.50 −3.50 × 170 with CDVA of 20/70. Although the patient had significant corneal astigmatism, they declined additional management options such as limbal relaxing incisions or laser refractive correction. Ocular motility was full, and there was no evidence of phoria or tropia. Intraocular pressures (IOP) were 14 mmHg in both eyes (OU). The pupil could dilate to ∼7 mm with dilation drops. Slit-lamp examination documented a clear cornea, irregular pupil, and aphakia OD. Posterior segment evaluation showed clear vitreous, normal disc, macula, and retina.

Given the absence of capsular support, options for visual rehabilitation were discussed, including an iris-claw lens or a scleral-fixated IOL. The patient was motivated to pursue an intraocular solution to eliminate his thick aphakic contact lens and improve his right eye's alignment with the dominant left eye with minimal residual anisometropia. We proposed the off-label use of a LAL with flanged intrascleral (Yamane) fixation to achieve the most precise refractive outcome. The goal was to maximize vision OD and align refractive status OU. Preoperatively, a trial lens simulation OD with target refractions of plano and −1.50 D demonstrated good patient tolerance; however, the patient preferred a distance target, and emmetropia was selected. After thorough counselling about the experimental nature of this approach, potential risks, and the need for multiple postoperative UV light adjustment sessions, the patient provided informed consent, with the plan to target emmetropia OD post LDD adjustment. Biometric data from the IOLMaster 700 is presented in Table 1. A +19.5 D LAL was chosen to achieve a target refraction of +0.01 using the Barrett Universal II formula. The patient provided written consent for the publication of this case report.

**Table 1:** Biometric data (IOLMaster 700).

Eye	Axial Length (mm)	Astigmatism (D)	Anterior Chamber Depth (mm)	Lens Status
OD	23.52	+4.43 @ 100°	NA	Aphakic
OS	22.98	+1.12 @ 136°	3.32	Phakic

## 3 Surgical technique

**Fig. 1:**
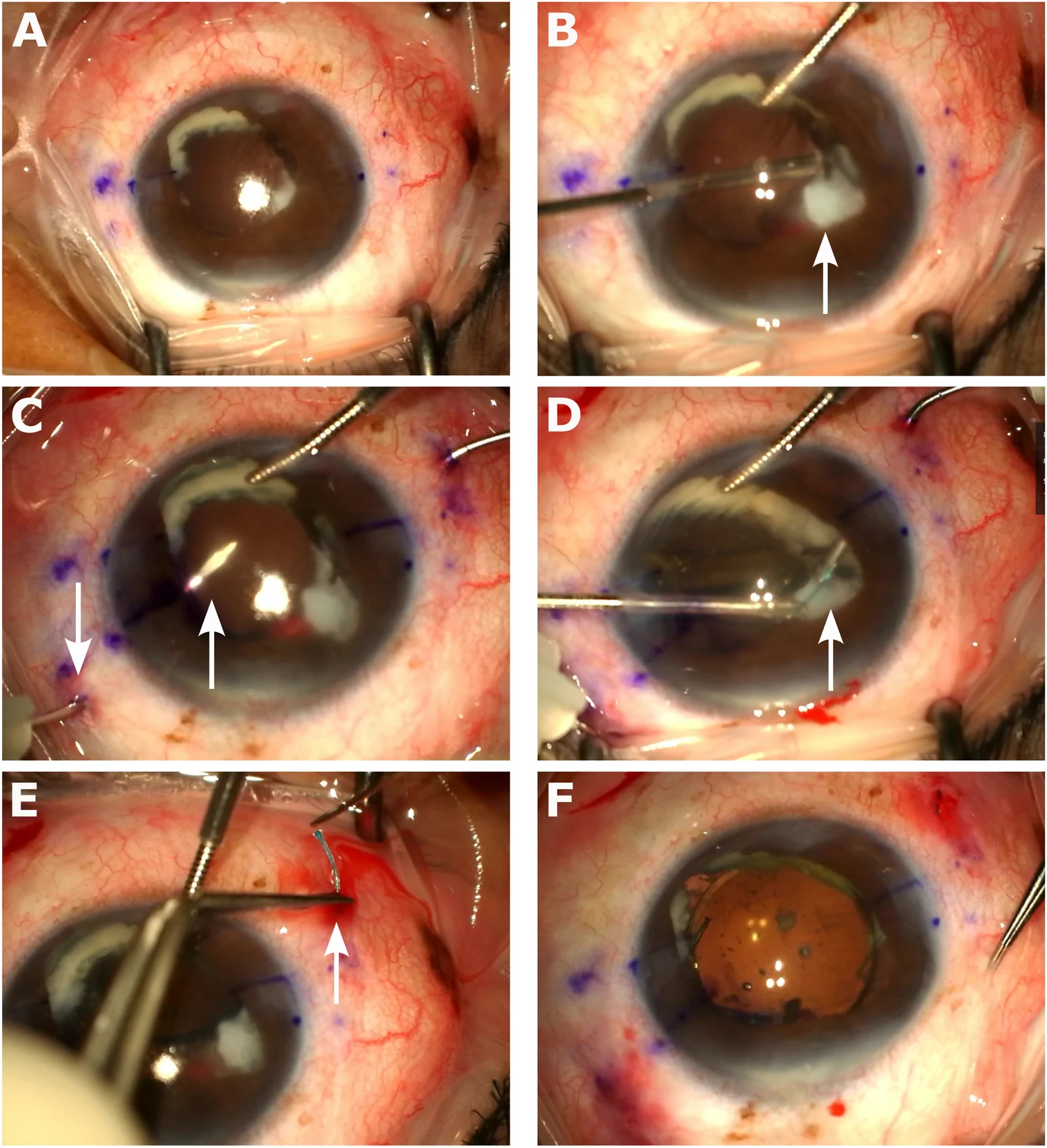
A. Intraoperative imaging of eye at baseline, B. Anterior vitrectomy removal of prolapsed vitreous and Soemmering's ring (white arrow), C. Formation of scleral tunnels using 27-gauge needles (white arrows), D. Haptic docking via the Yamane handshake (white arrow), E. Flange formation using cautery (white arrow), F. Intraoperative imaging of eye after intrascleral flanged fixation Glossary: intraocular lens (IOL), angle-supported anterior chamber IOLs (ASAC-IOLs), iris-fixated IOLs (IF-IOLs), and scleral-fixated IOLs (SF-IOL), Light Adjustable Lens (LAL), ultraviolet light treatment (LDD), right eye (OD), diopters (D), corrected distance visual acuity (CDVA), Intraocular pressures (IOP), both eyes (OU), left eye (OS), anterior chamber (AC), uncorrected distance visual acuity (UDVA), uncorrected near visual acuity (UNVA), effective lens position (ELP), polymethylmethacrylate (PMMA).

Preoperative scleral marking using India ink was placed 2.5 mm posterior to the limbus along the x-axis, with an additional 2 mm offset to guide future haptic externalization sites (Fig. 1A). Peribulbar anesthesia was administered, and an anterior chamber (AC) maintainer was used to preserve chamber depth throughout the case. Two side-port incisions were created at the x and y-axes with a 15° blade, and a superior main incision was fashioned using a 2.4 mm keratome. Anterior vitrectomy was performed to clear the prolapsed vitreous and remove residual Soemmering's ring spanning approximately 75° (Fig. 1B). Two 27-gauge needles were bent and introduced using the standard Yamane double-needle technique to create transconjunctival intrascleral tunnels (Fig. 1C). A +19.5 D LAL was then inserted into the anterior chamber. The leading haptic was docked using a Yamane handshake and externalized, followed by the trailing haptic, which was delivered similarly using micrograspers (Fig. 1D). Both haptics were flanged with low-temperature cautery to secure the IOL in the posterior chamber (Fig. 1E). A no-touch technique was employed to minimize optic handling. Inspection confirmed IOL centration and minimal tilt (Fig. 1F). The anterior chamber was reformed, incisions were hydrated and confirmed watertight, and the eye was patched at the end of the procedure. Following Yamane flanged scleral fixation of the LAL, 4 LDD treatments were performed over the course of 14 weeks. Postoperative outcomes including CDVA, uncorrected near visual acuity (UNVA), and refraction are presented in Table 2. Throughout follow-up, the LAL remained well centered with no complications such as cystoid macular edema or IOP elevation. The anterior segment was stable with a persistently irregular, nonreactive pupil, and the posterior segment remained healthy.

**Table 2:** Early postoperative outcomes.

Timepoint	CDVA	UDVA	UNVA	Refraction
Post-op Day 6	NA	20/250	NA	NA
Post-op Week 5	20/70	20/200	20/250	+1.50 -3.50 x 170
Post-op Week 8	20/40^−2^	20/100^−2^	20/100	+1.00 -3.25 x 170
Post-op Week 10	20/70	20/70^−2^	20/100	+0.75 -2.50 x 175
Post-op Week 14	20/40^−2^	20/70^−2^	20/63	0.00 -2.50 x 180

## 4 Discussion

This case demonstrates integration of two advanced technologies: the LAL and Yamane flanged intrascleral fixation in a case of post-traumatic aphakia and lack of capsular support. The Yamane approach with the LAL IOL enabled both stable anatomic fixation and precise postoperative refractive adjustment using LDD. These objectives are particularly important in the absence of the capsular bag, where prediction of the effective lens position (ELP) is inherently less reliable.

The Yamane technique provides a secure posterior chamber IOL fixation without dependence on capsular or iris support.^9^ Compared with AC-IOLs, it avoids direct contact with the corneal endothelium and angle structures, reducing the risk of endothelial cell loss, secondary glaucoma, and uveitis–glaucoma–hyphema syndrome.^10^ Similarly, it eliminates the need for iris tissue, which in this patient had been compromised by prior trauma, reducing the likelihood of iris chafing, pigment dispersion, and chronic inflammation compared to an IF-IOL.^11^ The sutureless nature of the Yamane technique also mitigates the risk of late IOL dislocation caused by suture breakage or erosion.^12^ In this case, flanged intrascleral fixation created a stable platform for the LAL in the absence of capsular and zonular support.

Our results align with prior reports demonstrating the Yamane technique's effectiveness. Large case series have reported dislocation rates of approximately 0 to 5 percent for Yamane-fixated IOLs compared with up to 28 percent for 10-0 prolene-sutured posterior chamber IOLs, and significant tilt in only 0 to 1 percent of eyes.^11^, ^12^, ^13^, ^14^ By placing the IOL in a near-physiologic location without sutures, the technique reduces anterior segment complications such as corneal touch and iris chafing. Besozzi et al., 2021 reported predictable refractive outcomes and no significant complications at six months using a standardised Yamane approach.^15^

Although Yamane fixation of the LAL has been described in isolated cases of Ectopia Lentis and Marfan syndrome, our report expands its use to post-traumatic aphakia. Margulies et al., 2023 reported favorable outcomes in a Marfan patient undergoing Yamane fixation of the LAL, followed by postoperative LDD adjustment to achieve micro-monovision.^5^ Another case by Ma et al., 2023 demonstrated safe LAL implantation via a trocar-assisted Yamane approach in a patient with ectopia lentis, with excellent refractive precision and visual acuity.^4^

The LAL allows refractive power adjustment postoperatively through targeted ultraviolet light modulation, mitigating the impact of ELP unpredictability. Refractive accuracy with secondary scleral-fixated IOLs remains limited, with approximately 31-50% of eyes achieving a result within ± 0.50 D of target, compared with 66-72% of eyes following in-the-bag implantation.^9^^,^^16^^,^^17^ In contrast, clinical trials using the LAL demonstrated that over 90% of eyes achieved a final spherical equivalent within ± 0.50 D of target following adjustment.^18^^,^^19^ In this patient, postoperative LDD refined refraction to 0.00 -2.50 x 180, optimizing binocular balance with the fellow eye. Such precision is unlikely to be achieved with a fixed-power scleral-fixated IOL.

The combination of Yamane fixation and the LAL may be applicable in other high-variance ELP contexts, such as scleral fixation after penetrating keratoplasty, secondary IOL implantation in adults with complex pediatric cataracts, or aphakia following vitreoretinal surgery. Successful application will depend on surgical proficiency in both the Yamane technique and the LAL adjustment protocol, careful patient selection, and adherence to postoperative follow-up requirements.

This report has several important limitations. Firstly, the LAL haptics are made from polymethylmethacrylate (PMMA), and their long-term stability under intrascleral fixation and repeated ultraviolet light treatments remains uncertain. Potential late complications include flange erosion, haptic deformation, or progressive IOL tilt. The effect on endothelial cell density over time was not assessed in this case and warrants future evaluation. Moreover, the LAL requires excellent centration and adequate pupillary dilation for effective UV adjustments; while this was achieved here, eyes with more extensive trauma, anterior segment scarring, or irregular pupils may present greater challenges. In addition, the long-term response of traumatized scleral tissue at flange sites remains unknown, raising the possibility of altered healing or late instability in eyes with prior injury. The efficacy of the LAL also depends on strict adherence to multiple postoperative adjustment visits and protection from unintended UV exposure, which may be difficult for some patients. Finally, the high cost and limited availability of the LAL currently restrict its accessibility, limiting broader adoption. Future studies are needed to clarify long-term safety, durability, and cost-effectiveness of this combined approach.

In summary, this case demonstrates the feasibility of Yamane flanged intrascleral fixation of an LAL for post-traumatic aphakia without capsular support, combining stable fixation with postoperative refractive refinement to address the longstanding challenge of unpredictable target refraction in secondary IOL implantation. Postoperative outcomes through week 14 indicate a favorable safety profile with progressive visual improvement. These findings highlight the role of the LAL in expanding the refractive precision of secondary IOL surgery, particularly in eyes with high anatomic complexity.

## CRediT authorship contribution statement

**Korolos Sawires:** Writing – review & editing, Writing – original draft, Visualization, Validation, Supervision, Software, Resources, Project administration, Methodology, Investigation, Funding acquisition, Formal analysis, Data curation, Conceptualization. **Dana Toameh:** Writing – original draft, Methodology, Investigation, Formal analysis, Data curation, Conceptualization, Resources, Software, Validation. **Raj Maini:** Writing – review & editing, Validation, Methodology, Investigation. **Sohel Somani:** Writing – review & editing, Writing – original draft, Visualization, Validation, Supervision, Software, Resources, Project administration, Methodology, Investigation, Funding acquisition, Formal analysis, Data curation, Conceptualization.

## Patient consent

Consent to publish this case report has been obtained from the patient in writing.

## Meeting presentation

None.

## Data sharing statement

Available upon reasonable request to the corresponding author.

## Claims of priority

After conducting a literature review on Sept 1, 2025, utilizing Medline, Embase, and CENTRAL with the keywords “Yamane technique,” “intrascleral flanged fixation” “intraocular lens implantation,” “light adjustable lens,” “sutureless intrascleral fixation,” and “traumatic aphakia” we did not identify any prior reports specifically describing the use of a intrascleral fixated LAL in a patient with post-traumatic aphakia.

## Use of generative artificial intelligence

None.

## Funding/support

None.

## Declaration of competing interest

The authors declare that they have no known competing financial interests or personal relationships that could have appeared to influence the work reported in this paper.
